# Comparative pangenomics unveils distinct host adaptation trends and conserved biosynthetic potential in microbiome *Clostridia*

**DOI:** 10.1093/molbev/msag247

**Published:** 2026-10-01

**Authors:** Lucas De Vrieze, Jan Aerts, Joleen Masschelein

**Affiliations:** Laboratory for Biomolecular Discovery & Engineering, Department of Biology, KU Leuven, 3001 Heverlee, Belgium; VIB Center for Microbiology, VIB, 3001 Heverlee, Belgium; Augmented Intelligence for Data Analytics, Department of Biosystems, KU Leuven, 3001 Heverlee, Belgium; Laboratory for Biomolecular Discovery & Engineering, Department of Biology, KU Leuven, 3001 Heverlee, Belgium; VIB Center for Microbiology, VIB, 3001 Heverlee, Belgium

**Keywords:** pangenome analysis, *Clostridia*, host adaptation, phylogenetic analysis, secondary metabolism, biosynthetic gene clusters, mobilome analysis

## Abstract

To thrive in diverse ecological niches, bacteria adopt various lifestyles that range from living freely in the soil to forming close associations with human and animal hosts. However, the impact of these adaptation processes on their genomes and metabolisms remains largely unexplored beyond the genus level. Investigating these evolutionary dynamics at higher taxonomic levels can enhance our understanding of the relationship between host adaptation and functional capabilities. Here, we examine the evolutionary trajectories and metabolic capabilities of the *Clostridia* class, which displays a wide variety of lifestyles and is of high importance for industry, medicine, and microbiome research. First, we uncover that the clostridial orders have significantly different genomic divergence rates. Second, we show contrasting host adaptation tendencies in *Oscillospirales* and *Lachnospirales*, the two clostridial orders dominated by host-associated species. Species of the former have often undergone extensive genomic and functional specialization toward a host-associated lifestyle, while species of the latter tend to show a lower level of host adaptation, retaining a remarkably high number of free-living trait genes and a high degree of metabolic versatility. Third, we reveal substantial differences in genomic architecture and metabolic versatility between the clostridial orders and link these to the progressing stages of host adaptation. Additionally, we identify widely conserved biosynthetic gene clusters, highlighting untapped biosynthetic potential of evolutionary significance. Hence, the beyond-genus level analyses in this study provide valuable new insights into bacterial adaptation with broad implications for evolutionary biology, microbiome research, and biotechnology.

## Introduction

Microorganisms continuously interact with their environment, adapting not only through short-term regulatory and metabolic responses, but also through long-term genomic changes. The continuous acquisition, mutation, and deletion of genes are closely tied to environmental selection pressures, giving rise to various lifestyles that vary in their dependence on external factors. These selection pressures drive evolutionary processes, such as host adaptation and genome streamlining, which can comprehensively reshape microbial genomes. This, in turn, has a profound impact on their functional and metabolic versatility, and hence their ability to survive in different ecological niches (Dufresne et al. 2005; Marais et al. 2008; Siguier et al. 2014).

Species belonging to the *Clostridia* class exemplify this evolutionary and ecological diversity. They exhibit a remarkable variety of lifestyles and phenotypes, ranging from thermophiles and psychrophiles to acidophiles and pathogens. They occupy diverse environments, including soil, sediments, and the intestinal tracts of humans and animals. This versatility underlies their increasing industrial and medical relevance, exemplified by industrially important species like *C. acetobutylicum* and *C. beijerinckii* (Roberts et al. 2010; Patakova et al. 2019; Birgen et al. 2021) and notorious pathogens like *C. botulinum*, *C. tetani*, and *C. difficile* (Smith et al. 2015; Cohen et al. 2017; Sandhu and McBride 2018). Moreover, *Clostridium* species were the first anaerobes shown to produce biosynthetic gene cluster (BGC)-encoded secondary metabolites with potent biological activities, reflecting their relevance for natural product discovery (Behnken and Hertweck 2012; Letzel et al. 2013; Sugimoto et al. 2019; Pahalagedara et al. 2020).

Despite the abundance, diversity, and relevance of *Clostridia*, few comparative studies have explored their evolutionary history and ecological adaptations beyond the species level that is traditional for pangenomics. Moreover, previous higher-level studies focused on only a few individual species or a single genus at most. For example, a study of *Clostridium* sensu stricto found evidence of a highly plastic genome, harboring a relatively large metabolic core and many specialized genetic capabilities that underpin the mainly free-living nature of this genus (Udaondo et al. 2017). Another study mainly focusing on *Clostridium* sensu stricto identified divergent evolution of the shikimate pathway in pathogenic clostridia and extensive mobility among their toxin genes (Cruz-Morales et al. 2019). Finally, a study of the gut-associated genus *Blautia* revealed that many gene exchange (GE) events involve metabolic functions, likely reflecting adaptation to the dynamic host environment, and suggested that such exchanges may have caused a metabolic dependence on other gut bacteria (Maturana and Cárdenas 2021). While informative, these studies do not provide broader class-level insights that allow cross-genus comparison.

In this work, we present a comprehensive study of the interplay between host adaptation, genome evolution and metabolic capacity for the taxonomic class of *Clostridia*. By combining pangenomic and phylogenetic analyses, we characterize the evolutionary dynamics within different orders, identify lifestyle trends from their current and ancestral genomic contents, and compare these trends to those inferred from ecological metadata. In parallel, we assess the metabolic versatility of each species and link this to their evolutionary trajectory. We also screened all genomes for a selection of genomic hallmarks and mobile genetic elements (MGEs) associated with progressing host adaptation. Finally, we zoom in on secondary metabolite BGCs, assessing their conservation and evolutionary dynamics across the class, and reveal biosynthetic features of potential ecological and biotechnological significance.

## Results

### Genome dataset composition

To examine how host adaptation has shaped genome architecture, metabolic potential and biosynthetic capacity in the *Clostridia* class, we first assembled a comprehensive dataset by downloading all available *Clostridia* genomes from NCBI. Following quality control and species-level dereplication, the final dataset comprised 1,010 genomes, sampled from various isolation sources, including open ecosystems as well as host-associated niches, such as human and animal microbiomes. After taxonomic classification using GTDB-tk, we selected four orders that together cover a large fraction of the *Clostridia* class while being sufficiently large for strong statistical power (n ≥30) (Add. File 2: Fig. S1): *Clostridiales*, *Lachnospirales*, *Oscillospirales*, and *Peptostreptococcales*. We also assigned each order a metadata-based reference lifestyle trend for comparison with the lifestyle trends inferred from our genomic analyses. Therefore, we retrieved ecological metadata for all species and classified them according to habitat and lifestyle. The *Clostridiales* and *Peptostreptococcales* comprise a mixture of free-living and host-associated species isolated from various environments, whereas the *Lachnospirales* and *Oscillospirales* are dominated by host-associated species isolated from animals and humans (Add. File 2: Fig. S2).

### Pangenome partition sizes and functional annotation indicate high genome plasticity

To investigate evolutionary dynamics, we first captured the gene conservation in all genomes by constructing a pangenome for the full genome dataset. Using a set of clustering and partitioning thresholds carefully selected after a parameter sweep (Add. File 3), the pangenomes for each assessed group of taxa were built and partitioned (Table 1). The recurringly large shell and cloud partitions suggest that there is a high level of diversity among all orders, meaning that each genome is likely to harbor unique gene families. This finding highlights the high level of genomic plasticity that is typical for species living in highly dynamic environments like open ecosystems or human-associated niches.

**Table 1:** Pangenome partition sizes.

	All genomes (n = 1,010)	*Clostridiales* (n = 209)	*Lachnospirales* (n = 349)	*Oscillospirales* (n = 164)	*Peptostreptococcales* (n = 94)
Core (>90%)	353 0.09%	690 0.79%	574 0.36%	465 0.59%	444 0.81%
Shell (0.1% to 90%)	158,627 39.78%	39,179 44.64%	63,580 40.06%	30,325 38.43%	21,451 39.04%
Cloud (<0.1%)	239,797 60.16%	47,903 54.58%	94,546 59.58%	48,109 60.98%	33,053 60.15%

Number of gene families in the pangenome partitions for each examined order, both in absolute numbers and relative to the total number of gene families in each order's pangenome.

After establishing the pangenome partitions, we examined the functional differences between the orders (Fig. 1). The functional profiles are similar for most core genomes and is dominated by ribosomal proteins (COG category J). A notable difference is the higher proportion of signal transduction genes in the core genome of *Clostridiales* (COG category T), characteristic of a free-living lifestyle that requires more complex environmental sensing and more specialized responses. In almost all orders, the majority of the shell and cloud gene families have no known function (COG categories S and –), suggesting the presence of MGEs, highly specialized functions, and/or rapidly evolving genes that fall outside the scope of our current understanding.

**Figure 1:**
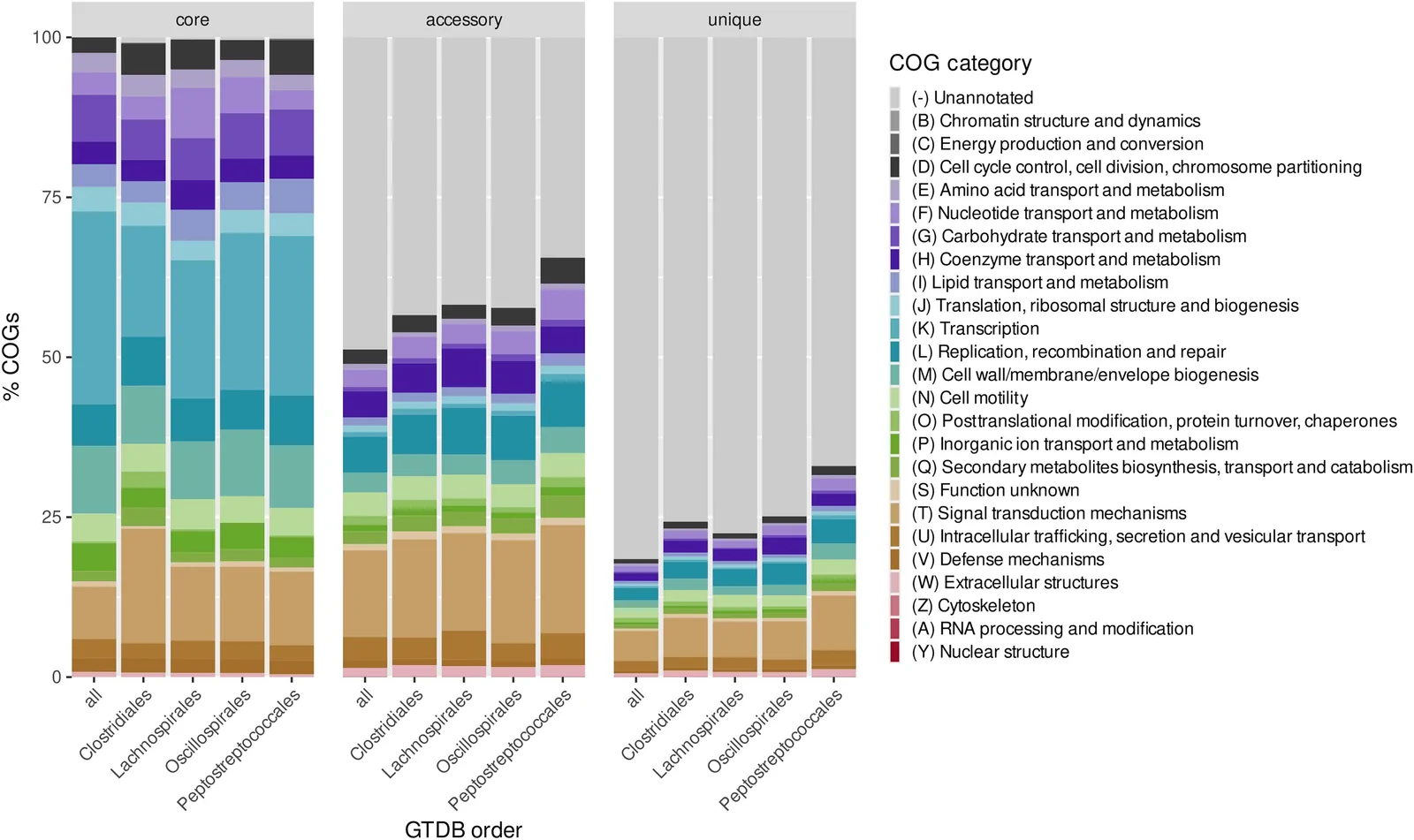
COG functional annotation profiles for all pangenome partitions of each examined clostridial order. Fractions are referenced to the number of gene families in a partition. The functional profiles are similar, except for a larger share of signal transduction genes in the core genome of the *Clostridiales* order, which is consistent with a free-living lifestyle. For the exact category fractions and counts, see Add. File 8.

### KEGG pathway completeness analysis uncovers two distinct levels of metabolic versatility

A KEGG pathway completeness analysis gauges the metabolic versatility of an organism by assessing the completeness of metabolic pathway modules (KEGG pathway modules) based on the genomic presence of their constituent genes. Remarkably, two distinct levels of pathway completeness were observed for the full genome dataset. Three hundred and ninety-four out of 1,010 organisms exhibit substantially higher pathway completeness, indicative of more versatile metabolic capabilities (Fig. 2). This difference is not driven by a limited number of pathways, but is the result of a genome-wide trend, as multiple modules tend to be more complete. This aligns with earlier reported observations of a remarkably high metabolic versatility within the *Bacillota* phylum, especially within the *Clostridium* genus (Vieira-Silva et al. 2016; Pascal Andreu et al. 2023). Multiple clostridial genera also contain both versatile and nonversatile species, confirming an intra-genus heterogeneity that has previously been reported (Vieira-Silva et al. 2016).

**Figure 2:**
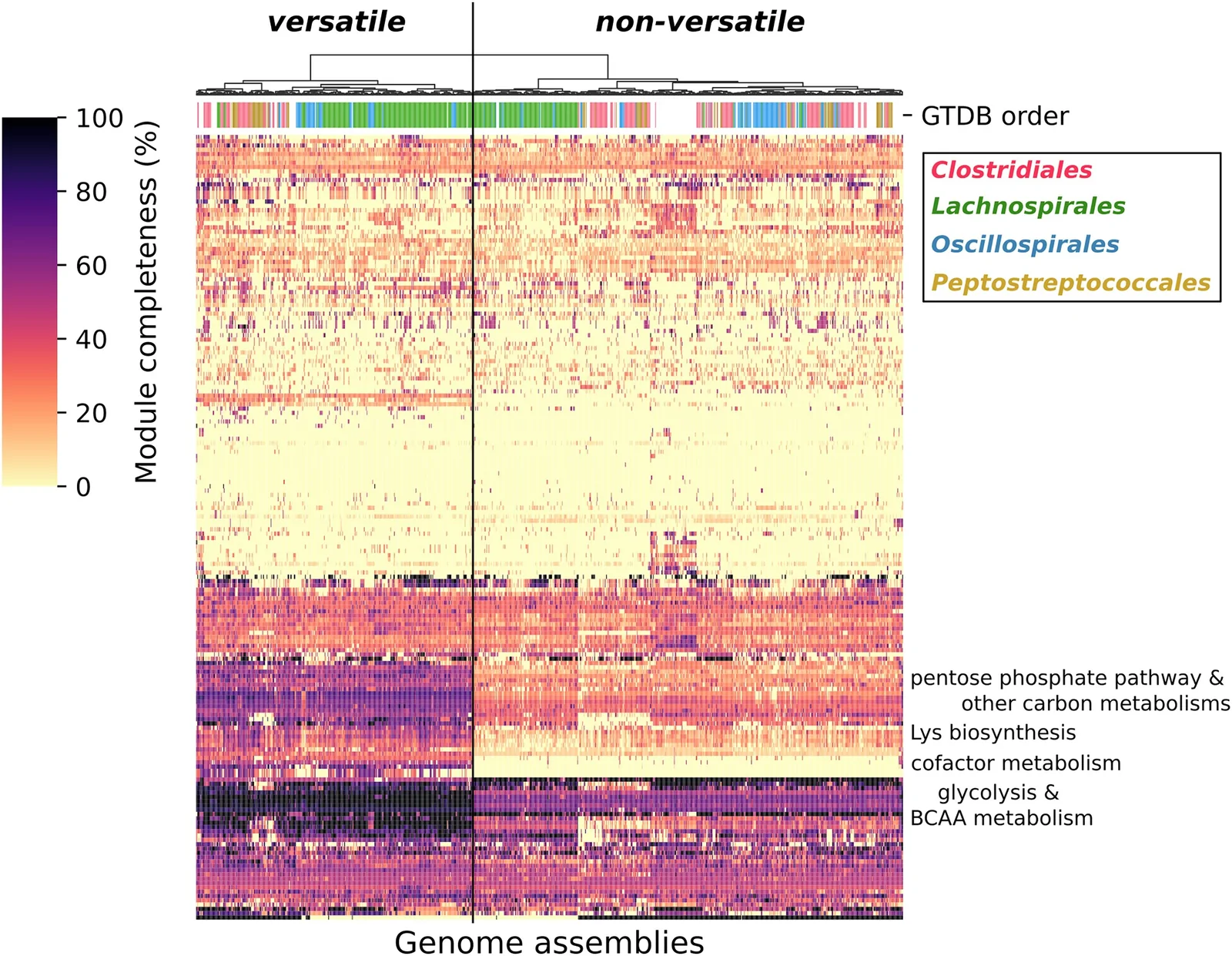
Ward's clustered heatmap of KEGG module completeness, annotated by taxonomic order. Metabolically versatile genomes recurringly show a general trend in higher module completenesses. In summary, the key differing modules comprise glycolysis-related and BCAA biosynthesis pathways, as well as Lys biosynthesis, the pentose phosphate pathway, and other carbon metabolisms. For a figure with all KEGG module labels, see Add. File 2: Figure S5.

Interestingly, except for the *Clostridiales* order, metabolic versatility appears to be independent of genome size and tends to concentrate within particular lineages. For example, notable *Lachnospirales* genera and lineages such as *Enterocloster*, *Lacrimispora*, *Butyvibrio*, and one of the two *Blautia* lineages are almost entirely metabolically versatile (Add. File 2: Fig. S4). Within *Oscillospirales*, metabolically versatile species are concentrated in the *Acutalibacteraceae*, *Ruminococcus*, and *Faecalibacterium* lineages, while versatile *Peptostreptococcales* genera are *Peptostreptococcus* and *Clostridioides*.

The most pronounced differences in module completeness between versatile and nonversatile species are found in core metabolic processes, including central carbon metabolism (glycolysis, pentose phosphate pathway) and branched-chain amino acid and Lys biosynthesis. The many metabolically versatile species found in host-associated environments thus have a higher degree of metabolic independence than typically expected for host-associated bacteria. This more developed primary metabolism may, in turn, support a more extensive secondary metabolism, providing additional adaptive advantages. The *Lachnospirales* order in particular contains a higher fraction of versatile species than the other orders (60% vs. 25% to 35%), revealing a stronger tendency for metabolic versatility than anticipated for an order dominated by host-associated species (Add. File 2: Fig. S3).

### Genomic divergence rate analysis reveals significantly different evolutionary dynamics

The higher metabolic versatility of the *Lachnospirales* suggests a distinct evolutionary trajectory compared to the *Oscillospirales* and *Peptostreptococcales* orders. To assess whether this indeed reflects a different trend in evolutionary dynamics, GE models were fitted for each order separately and statistically compared to each other using *Panstripe*.

*Panstripe* uses a compound generalized linear model (GLM) to relate phylogenetic branch lengths to the number of genes exchanged at those branches. Using an additional GLM term, internal nodes of the tree can be differentiated from terminal ones, yielding core and tip GE rate estimates, respectively. Exchange rates that are significantly different from zero signify an association between branch length and the number of GE events, and thus active GE. The core rate represents the long-term GE rate shaping the overall phylogeny structure, while the tip rate captures short-term genomic dynamics, such as transposon- or phage-mediated gene mobility. However, in highly diverse genome sets, such as the dataset analyzed here, orthologous gene families may have been split into multiple families during pangenome construction, artificially inflating both core and tip GE rates. Consequently, *Panstripe* models here a more general genomic divergence (GD) rate, that captures both intrinsic gene evolution as well as GEs, rather than only the latter. Nevertheless, we argue that this more general GD rate is well-suited to address our research question, as it provides a measure of the pace at which genomes have been evolving.

GD modeling revealed several interesting differences in evolutionary dynamics between the four orders (Fig. 3). First, the *Oscillospirales* have the lowest core divergence rate, which is consistent with the observation that streamlined genomes of host-associated species evolve at a reduced rate (Dufresne et al. 2005; Marais et al. 2008). In contrast, the *Lachnospirales* have significantly higher core and tip divergence rates, indicating a higher level of both long-term gene divergence and short-term gene mobility, respectively. This pattern is unexpected for an order dominated by host-associated species and may suggest that many *Lachnospirales* lineages are undergoing continued genomic adaptation to their host-associated lifestyles (see also Fig. 5g) (Siguier et al. 2014). The tip divergence rate of the *Peptostreptococcales* is higher as well, but the core rates are not. This observation may reflect a higher frequency of early host adaptation, which entails increased gene mobility, or alternatively, late host adaptation, in which long-term divergence has slowed down while residual gene mobility persists.

**Figure 3:**
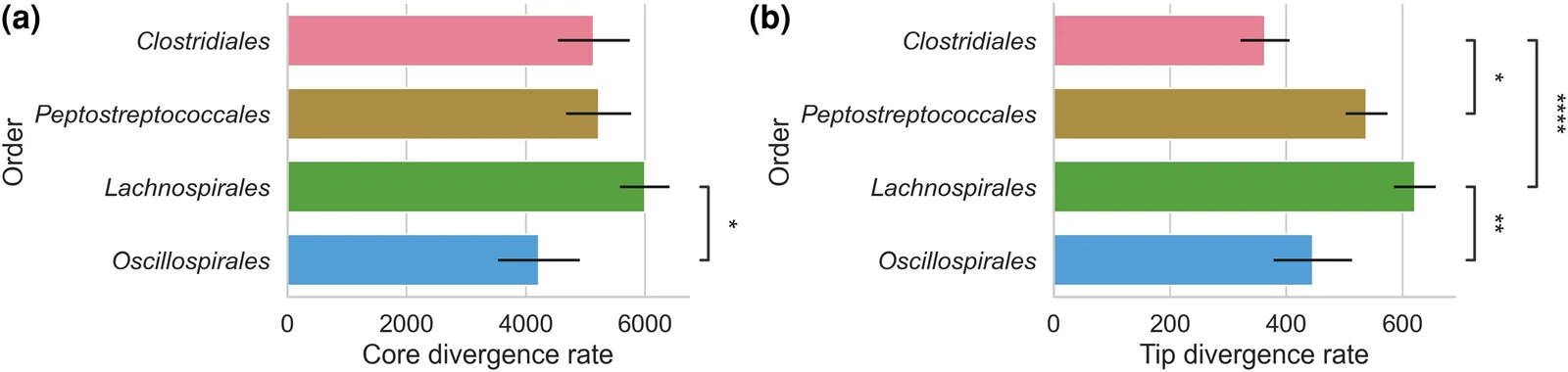
Divergence rate fits for each order. a) Core and b) tip divergence rates of the GD models for each order, including standard error and statistical significance annotations. *Oscillospirales* have a significantly lower divergence rate, consistent with the predominance of host-associated species, whereas *Lachnospirales* display a high divergence rate inconsistent with such a predominance. This inconsistency may be explained by *Lachnospirales* species tending to be in earlier stages of host adaptation than *Oscillospirales*.

### Ancestral state reconstruction suggests different ancestral lifestyles for host-associated-dominant orders

The metabolic versatility and GD analyses suggested differences in lifestyle preferences among the host-associated-dominant orders. To investigate whether these preferences may have been ancestrally inherited, we conducted an ancestral state reconstruction (ASR). An ASR identifies at each node the clade-specific genes that have been passed on from the ancestor, allowing gene gains, and losses to be traced along the phylogeny. Functional annotation of these genes can provide insight into the metabolic capabilities and potential lifestyles of each order's ancestor, and help determine whether current lifestyle preferences may have deeper evolutionary origins. Interestingly, the ASR of the clostridial phylogeny recurringly shows relatively high numbers of gene gain and loss events in the vicinity of the root node of each order (Fig. 4).

**Figure 4:**
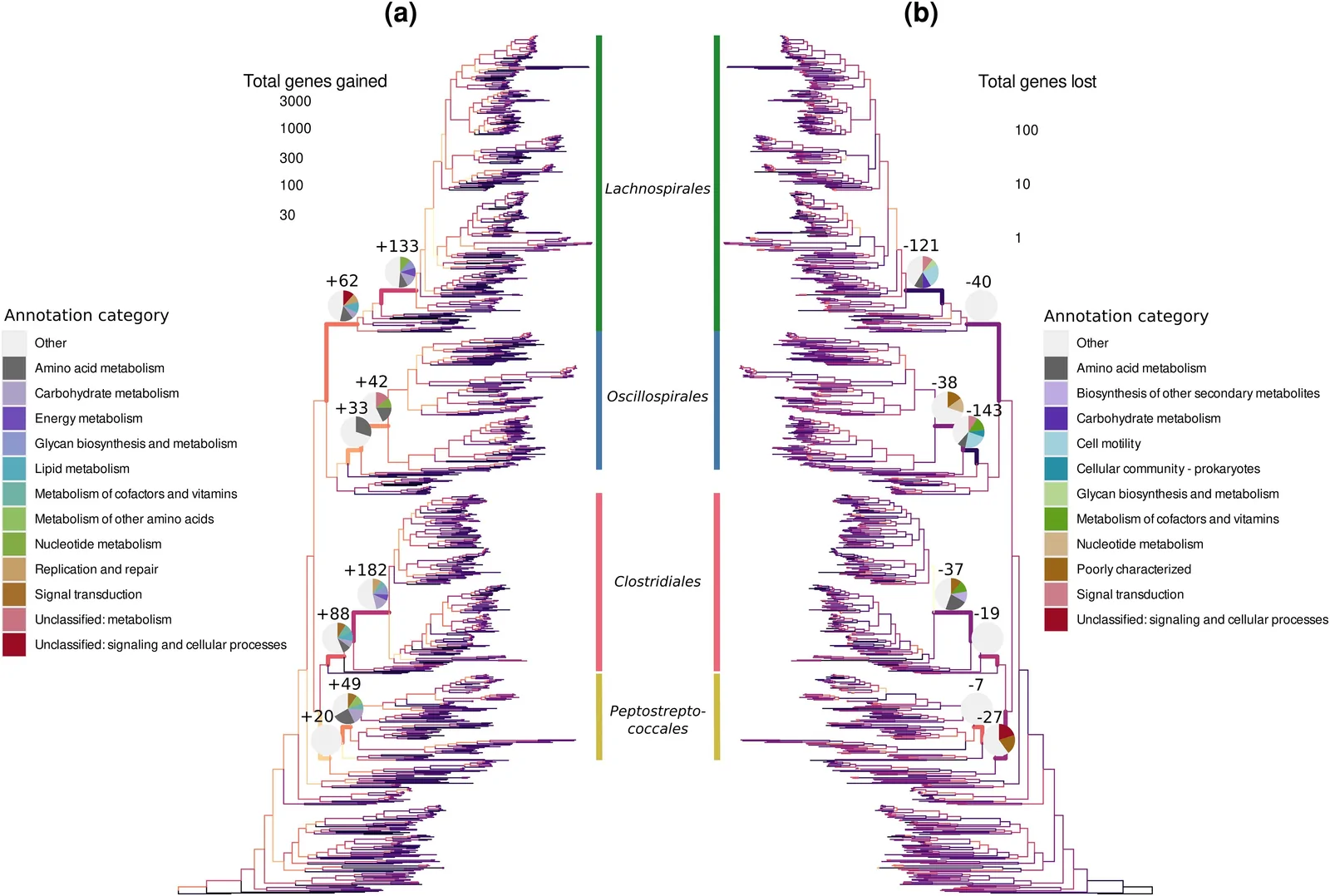
Wagner parsimony gene exchange profiles. a) Gene gain and b) loss profiles for the full genome set (colored branches), and absolute number and KEGG BRITE level B annotation profile of exchanged genes for a selection of ancestral nodes (inset pie charts). For each node, only the top five categories are shown, excluding the “Unannotated” category, each shown category requiring at least three counts.

A total of 796 genes were present in the last common ancestor (LCA) of the *Clostridia*. These are associated with core cellular processes like glycolysis, tRNA biosynthesis and the ribosomes. In addition, they include genes for flagellar assembly, two-component signaling systems and DNA repair systems, suggesting that the LCA of the clostridial lineage was a well-equipped free-living microbe.

Analysis of the most recent common ancestors (MRCA) of each individual order interesting differences in both the number and functional annotation of the exchanged genes (Add. File 5). The MRCA of *Clostridiales* gained 88 genes, only half of which could be functionally annotated. These are mostly associated with metabolism, signal transduction, and cell motility, consistent with a free-living lifestyle. In contrast, this MRCA lost only 19 genes, of which the annotated fraction is again mostly metabolism-related. Notably, the ancestor of a large subcluster containing the *Clostridium* sensu stricto clade has gained 182 genes, which are mostly associated with metabolism, and DNA replication and repair, indicating that this subcluster's ancestor even further refined and expanded its inherited free-living capacities.

The *Oscillospirales* MRCA gained 42 genes and lost 38, mostly related to metabolism in both cases. Interestingly, the direct ancestor of this node has lost a considerable number of gene families involved in cell motility, cofactor metabolism and signal transduction pathways. These losses align with the reduction in free-living capabilities that typically occurs when a microorganism transitions to a host-associated lifestyle.

In contrast, the *Lachnospirales* MRCA has gained 62 genes and lost 40. The acquired genes are associated with amino acid and lipid metabolism, and DNA replication and repair, while most lost ones lack functional annotation. Also here, a pronounced GE was observed in a descendant lineage corresponding to the MRCA of the historical *Clostridium XIVa* clade, which gained 133 genes and lost 121. Gained functionalities affect several metabolic systems, including secondary metabolite biosynthesis, while lost functionalities are linked with cell motility and signal transduction. Both the number of exchanged genes and the combination of metabolic expansion with the loss of free-living traits indicate that the *Lachnospirales* and the *Oscillospirales* followed different evolutionary trajectories toward host adaptation, which may, in turn, help explain the different lifestyle-associated patterns observed nowadays.

Finally, the *Peptostreptococcales* ancestral lineages exchanged relatively few genes. For example, the MRCA gained 49 genes and lost only 7. The gained genes are involved in several metabolic subsystems, such as amino acid metabolism and glycan biosynthesis. Similarly to the GD analysis, this low level of GE may indicate that the *Peptostreptococcales* MRCA was either at the end of the host conversion process or had only just started it.

### Focused mobilome analysis confirms different levels of host adaptation

Both the GD and the ASR analyses indicate varying host adaptation trends among the clostridial orders. Moreover, the higher metabolic versatility observed in *Lachnospirales* may be related to lower levels of host dependence. To further substantiate these inferred stages of host adaptation, we screened the entire genome dataset for the abundance of genomic hallmarks associated with progressing host adaptation.

Specifically, the transition from a free-living to a host-associated lifestyle in bacteria is frequently coupled with a genome streamlining process driven by insertion sequences (ISes) and intrinsic mutations (Siguier et al. 2014). ISes are a type of intracellular MGEs that employ transposases for their mobility. When bacteria move from a nutritionally poor free-living environment to a nutrient-rich host-associated niche, many genes become redundant due to the constant availability of metabolites. This causes a reduced selection pressure on these nonessential genes, enabling higher mutation rates and IS insertions, which, together, drive pseudogene formation (Marais et al. 2008). Over time, the accumulation and multiplication of IS regions promotes genome rearrangements and deletions, ultimately yielding smaller streamlined genomes tailored to new host-associated lifestyles (Fig. 5g) (Siguier et al. 2014). Note that this commensal genome streamlining process is fundamentally different from the one occurring in free-living organisms (Giovannoni et al. 2014).

**Figure 5:**
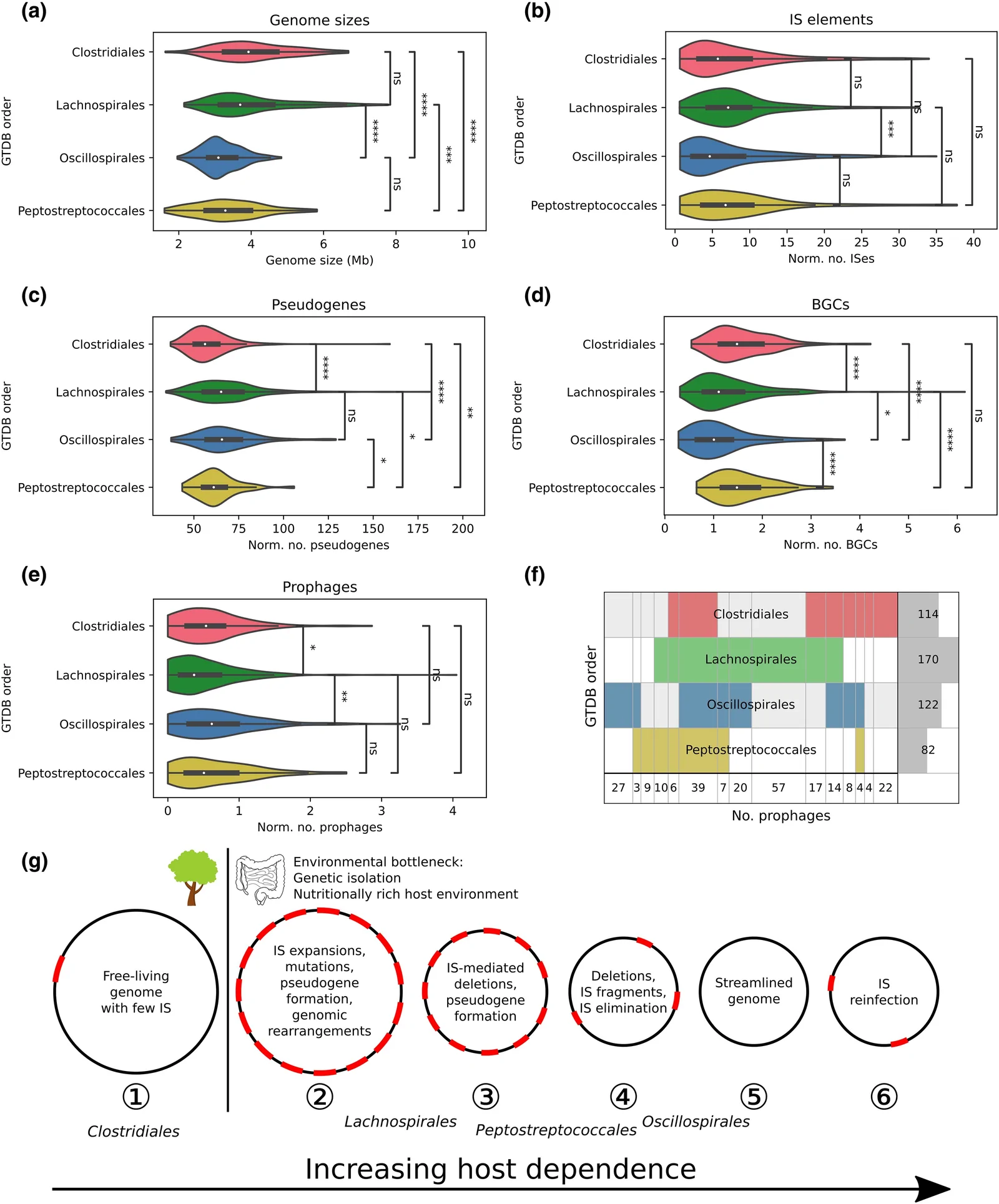
Screening of the genomic hallmarks of host dependence. a–f) Comparison of the abundance of a genomic feature across all orders. Feature counts are normalized by dividing by the genome length in Mb, and statistically compared using pairwise Brunner–Munzel tests with Bonferroni correction (*α* = 0.05). a) genome size, b) insertion sequence elements, c) pseudogenes, d) biosynthetic gene clusters, e) prophages, f) GTDB taxonomic order of the prophage hosts. g) Schematic overview of the impact of the genome streamlining process on the bacterial genomic architecture, including the most likely position of a species of each clostridial order in this process. Figure inspired on (Siguier et al. 2014). (1) The (ancestral) free-living genome has only a few ISes. (2) As a result of an environmental bottleneck, IS expansion occurs, leading to an increased abundance of ISes, pseudogenes and mutations. Genomic rearrangements occur. (3) With time, the repetitive regions of the ISes facilitate the deletion of non-essential genomic regions. (4) Eventually, extensive deletion leads to the elimination of the ISes. (5) Finally, the genome reaches its streamlined form, with a gene content tailored for life in its host environment. (6) Upon contact with IS-carrying strains or phages, ISes may be reintroduced.

To test this hypothesis, we screened the complete genome dataset for the abundance of multiple MGEs and genomic features associated with host adaptation: (1) genome sizes, (2) ISes, (3) pseudogenes, (4) prophage regions, and (5) BGCs. (1), (2), and (3) are established hallmarks of the genome streamlining process. (4) may reflect a shift in environmental niche. (5) may provide an indication of metabolic versatility, as it depends on the availability of diverse precursor metabolites.

The results align strikingly well with our previous interpretations. First, *Oscillospirales* and *Peptostreptococcales* genomes are significantly smaller than those of *Clostridiales* and *Lachnospirales* (Fig. 5a).

Second, *Lachnospirales* and *Peptostreptococcales* genomes harbor significantly more ISes than those of *Clostridiales*, which, in turn, contain more than *Oscillospirales* genomes (Fig. 5b). This pattern is consistent with the hypothesis that *Lachnospirales* species tend to be in the early stages of host adaptation during which IS expansion may accompany ongoing genomic restructuring. In contrast, the smaller genomes and fewer IS elements in *Peptostreptococcales* suggest that species within this order tend to be at a slightly more advanced stage of the host transition process than the *Lachnospirales*.

A more detailed analysis of the IS families revealed some substantial differences in their distribution across the clostridial orders (Add. File 2: Fig. S6). For example, family IS1634 is only present in *Lachnospirales* and *Peptostreptococcales*, while family IS5 is only found in *Oscillospirales*. Notably, families IS110, IS66 and ISL3 are more prevalent in *Lachnospirales* species. As these IS families do not have a specific insertion site preference (Siguier et al. 2014, 2015), their abundances may merely be the result of IS expansion during the host conversion process.

Third, pseudogenes are significantly less abundant in *Clostridiales* than in *Peptostreptococcales,* which in turn harbor less than *Oscillospirales* and *Lachnospirales.* (Fig. 5c). This implies that species in both host-associated-dominant orders are more likely to have undergone or be undergoing intense pseudogene formation, which is consistent with progressive genome streamlining during host adaptation.

Fourth, the number of secondary metabolite BGCs is significantly higher in *Clostridiales* and *Peptostreptococcales*, with *Lachnospirales* and *Oscillospirales* at comparably lower levels (Fig. 5d). *Clostridiales* and *Peptostreptococcales* species are thus more likely to maintain a greater capacity for specialized metabolism than species of the other two orders. Given that other genomic features indicate that the majority of the *Lachnospirales* species are at an early stage of host adaptation, this observation may imply that BGCs are among the first genomic features to be eliminated, even when metabolic versatility is maintained. Alternatively, this may reflect current limitations in the detection and annotation of BGCs from host-associated species. Analysis of BGC classes revealed that the most substantial differences in abundance occur among the cyclic lactone autoinducer, NRPS(-like), and RiPP-like classes. In addition, there is a consistent presence of ranthipeptides and terpene precursors across all orders (Add. File 2: Fig. S7).

Finally, *Lachnospirales* genomes contain on average significantly less prophages than those of the other orders (Fig. 5e). In terms of host range, however, *Lachnospirales* carried the highest diversity in prophage types, while *Peptostreptococcales* had the lowest. Interestingly, about 75% of the detected prophage types were found in clostridial species that were part of different orders, with 39 occurring across all four orders (Fig. 5f). In addition, some prophages carry passenger genes for methyltransferases, restriction enzymes, toxin(-antitoxin system)s, and transporters (including beta-lactam efflux pumps and other resistance proteins), highlighting a mechanism by which clostridial species may acquire new genes and adaptive traits (Add. File 6). Although clear differences in prophage abundance and diversity were observed, it remains unclear whether this merely reflects a niche shift or whether these prophage-encoded genes that have become essential for the bacterium's survival due to functional integration.

In conclusion, the abundance of these genomic features further supports the hypothesis that species from different clostridial orders tend to be at different stages of the host adaptation process (Fig. 5g).

### BGC clustering analysis reveals strongly conserved BGCs

The species in our clostridial dataset have faced diverse ecological and evolutionary selection pressures. Hence, BGCs that have remained conserved across these varying conditions likely encode functions that offer substantial evolutionary advantages that justify their long-term conservation. Such high-potential BGCs are expected to emerge in BGC clustering analyses.

Our clustering analysis revealed that the five largest BGC groups consist of two terpene-precursor groups and three ranthipeptide groups. Both *Clostridiales* and *Lachnospirales* harbor one terpene-precursor and one or two ranthipeptide BGC groups (Fig. 6). Alignment of representative gene clusters from the three largest ranthipeptide groups shows that the core biosynthetic genes, ie the SCIFF precursor and the RRE gene, are conserved across all groups (Add. File 2: Fig. S8). Moreover, these ranthipeptide BGCs are well-distributed across the phylogeny, with each species encoding maximum one ranthipeptide (Add. File 2: Fig. S9). Interestingly, two of these ranthipeptide BGCs have been experimentally shown to participate in quorum sensing activities (Chen et al. 2020). The fact that these two BGCs belong to the same ranthipeptide group in our analysis suggests that similar quorum sensing functions may be associated with this entire ranthipeptide group.

**Figure 6:**
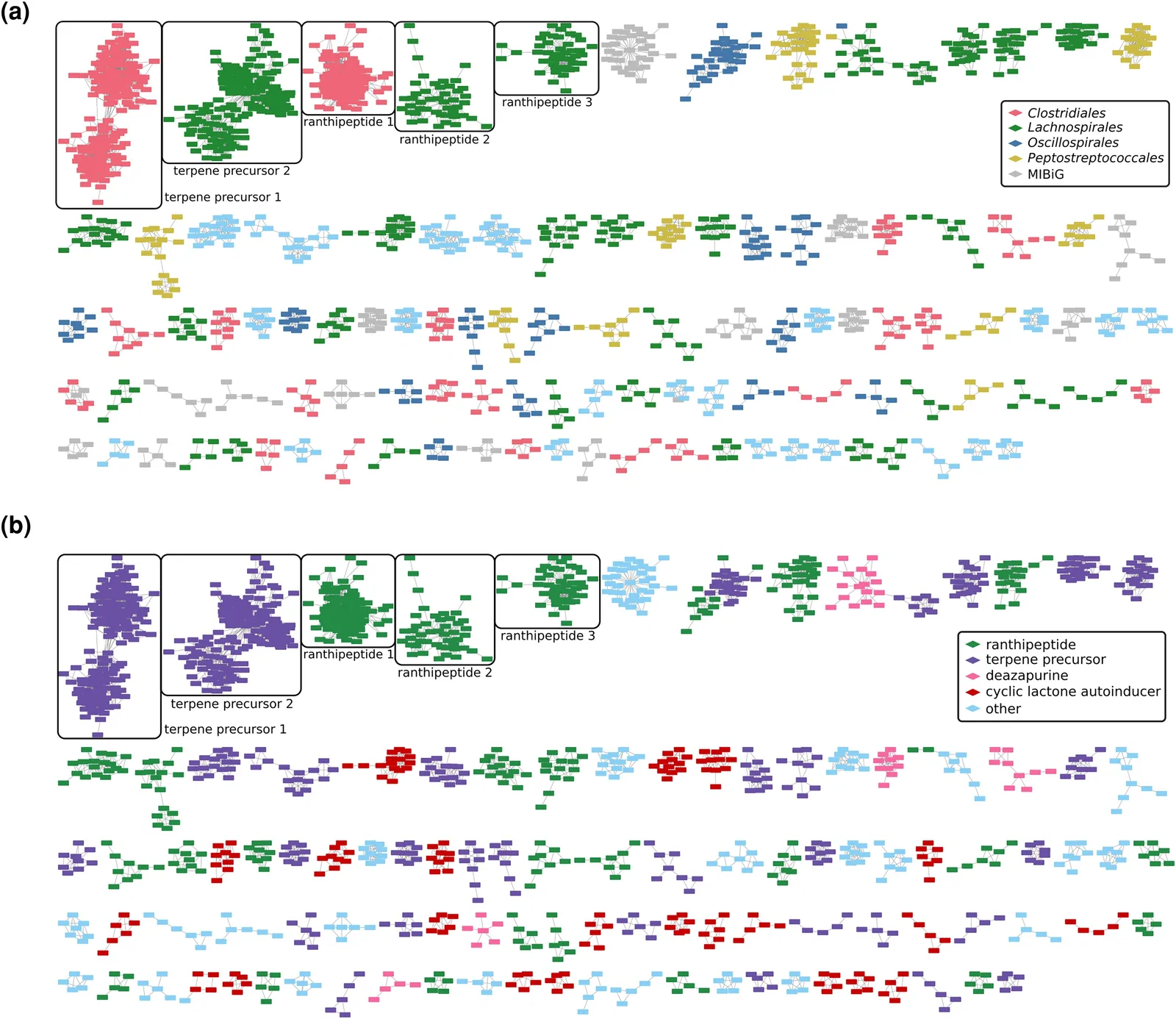
BiG-SCAPE similarity networks of all BGCs detected in our full genome set (threshold 0.3), annotated a) by clostridial order or as MIBiG entry, and b) by BGC type. The five largest groups on the top left are either terpene precursors or ranthipeptides. Only groups with at least five members are shown.

The smaller identified BGC groups are predicted to encode cyclic lactone autoinducers and deazapurines, next to smaller ranthipeptides and terpene precursor groups. We co-clustered our BGC set with the MIBiG database, a highly curated and standardized repository of known BGCs (Zdouc et al. 2025). Interestingly, very few MIBiG BGCs co-cluster with our clostridial BGCs, underscoring the novelty of these conserved ranthipeptide and terpene precursor BGCs, and their high potential for functional significance. We also observed a large group of singleton BGCs (Add. File 7), which may comprise BGCs acquired more recently by horizontal gene transfer or gene rearrangement. While these singletons have not undergone sufficient evolutionary selection to refine their functional significance, the possibility that they encode potent and biologically relevant activities cannot be excluded.

## Discussion

This work presents the first comparative pangenomics analysis of multiple orders of the *Clostridia* class and reveals intriguing differences in their host adaptation tendencies. Moreover, it uncovered a remarkably large repertoire of uncharacterized BGCs, including several highly conserved ranthipeptide families, highlighting substantial untapped biosynthetic potential.

A major finding of this study is that species from different clostridial orders tend to occupy different stages along the host adaptation process. By combining multiple independent genomics approaches, we identified the stage of host adaptation that appears most prevalent within each order. In particular, the tendency of *Lachnospirales* species toward earlier stages of host adaptation adds an important nuance to their commonly assigned ecological status as host-associated gut symbionts. Much of the genomic and metabolic diversity of this clade is obscured by the generic label “host-associated.” Bacteria in the early stages of host association likely still retain part of their ancestral free-living gene repertoire, a hypothesis supported by our KEGG pathway completeness assessment, which revealed a tendency for higher metabolic versatility among *Lachnospirales* species. The combination of their higher innate capacity to produce more complex metabolites and their adaptation toward host environments positions these species as attractive starting points for the development of anaerobic chassis organisms for heterologous expression of complex biosynthetic pathways. Moreover, their ecological compatibility with the gut environment suggests a high potential for therapeutic applications, such as delivery of bioactive compounds or engineered functions in gut microbiomes (Kubiak and Minton 2015).

Another key outcome of this study is the comprehensive identification and comparative analysis of clostridial BGCs, providing, to the best of our knowledge, the first comparative view of the natural product repertoire beyond PKS and NRPS classes across multiple clostridial orders. Despite extensive host adaptation and a long track record of exposure to varying selection pressures, several ranthipeptide and terpene precursor BGCs have been widely conserved. The lack of MIBiG entries co-clustering with these BGCs underscores their novelty, tapping further into the unexplored richness of BGCs from the anaerobic realm. These novel conserved BGCs are prime targets for further experimental characterization.

Nevertheless, several considerations should be taken into account when interpreting these findings. First, the lifestyle trends and preferences observed in this study represent general tendencies within each order and should not be extrapolated to individual species without further analysis. For example, individual *Lachnospirales* species may already be highly adapted to their host due to stronger selection pressures, or because they started the host adaptation process early compared to their close relatives.

Second, we aimed to incorporate as much diversity as possible in our dataset. However, as the amount of available genomic data differs among orders, the resulting dataset is taxonomically imbalanced. Overrepresented lineages may therefore bias analysis outcomes and obscure diversity in other lineages. Such taxonomic imbalance comes in two forms: (1) unequal representation of species within the available genomic data (“known imbalance”) or (2) the available data themselves being an imbalanced sample of natural diversity (“unknown imbalance”). To mitigate (1), we performed a full-genome dereplication of our genome dataset by Average Nucleotide Identity (ANI), although this inevitably comes at the cost of losing strain-specific diversity in the discarded genomes. To mitigate (2), we challenged our findings against different imbalance levels using differently sized random subsamples from our original dataset. All outcomes were robust, except for the ISE and the GD findings. However, in both cases, a follow-up effect size analysis using Hedges' g values indicated that the subsampling likely reduced statistical power and obscured a genuine signal rather than revealing a bias caused by taxonomic imbalance (Text S1).

Third, the pangenome is fundamental to both the GD and the ASR analyses, and its composition depends heavily on the clustering and partitioning thresholds applied. We mitigated this strong dependency by (1) performing an extensive parameter sweep to optimize these threshold values (Add. File 3), (2) corroborating the pangenome-dependent findings with pangenome-independent analyses, such as the mobilome screening, and (3) successfully verifying the robustness of the GD analysis results across different pangenome threshold values (Text S1, Add. File 10).

Finally, we deliberately applied a feature-first approach, inferring lifestyle trends from genomic features previously associated with host adaptation in the literature, rather than a metadata-first approach that would identify distinctive genomic features based on an ecological metadata set. This choice may introduce a bias, because our selected features were manually selected based on their established relevance to host adaptation mechanisms (Marais et al. 2008; Giovannoni et al. 2014; Siguier et al. 2014). In contrast, a metadata-first approach typically leaves this selection to a machine learning classifier. However, a metadata-first approach would itself be limited by the ambiguity of the available ecological metadata. For example, the isolation site, a commonly used variable, does not necessarily reflect the microbe's habitat and lifestyle, which is particularly relevant for the numerous spore-forming species within *Clostridia*. Moreover, categorical variables such as host association and pathogenicity are better interpreted as continuous spectra rather than distinct classes, as illustrated by the existence of host adaptation processes and opportunistic pathogenicity. This ambiguity undermines the reliability of metadata-based categorization and, consequently, metadata-first inference. Therefore, in this study, ecological metadata served only as a secondary information source to corroborate the genomically inferred host association tendencies.

To conclude, the combined application of comparative genomics, phylogenetic techniques, and targeted analysis of genomic features at higher taxonomic levels offers a flexible approach to investigate and compare host adaptation trends across different taxonomic clades. We uncovered counterintuitive differences in host adaptation tendencies between distinct clostridial clades dominated by host-associated species, which reflected in their genome architectures and metabolic versatility. Taken together, the findings of this study are an encouraging starting point for the systematic investigation of adaptation levels in other “host-associated” bacteria classes than *Clostridia*. Moreover, in the light of natural product research, our findings pave the way for (1) establishing clostridial species as new anaerobic host organisms for a diverse range of applications, as well as for (2) expanding the natural product repertoire further into the anaerobic realm.

## Materials and methods

### Data gathering, QC and filtering

All genome assemblies were retrieved from RefSeq using the search query “Clostridia” in the NCBI Datasets CLI (v16.2.0) (O’Leary et al. 2024), excluding MAGs and atypical assemblies (n = 10,599 as of 2025 May 22). The resulting set was filtered by number of contigs (≤200), N50 (≥50 kb), completeness (≥90%), and contamination (≤10%), and dereplicated at species level using skDER (v1.3.1) (Salamzade et al. 2025) (ANI cutoff: 96%, aligned fraction cutoff: >50%; Add. File 2: Fig. S11), resulting in 1,010 representative assemblies. For consistency, all assemblies were reannotated with Bakta (v1.9.1) (Schwengers et al. 2021) and taxonomically verified using GTDB-tk (v2.4.1) (Chaumeil et al. 2022). To ensure sufficient statistical power in downstream analyses, only taxonomic orders with at least 30 representative species were retained.

### Ecological metadata retrieval and classification

The ecological metadata were retrieved for all selected assemblies using the BV-BRC CLI (v1.048). Lifestyle and habitat classifications were inferred and curated manually from the existing columns in the metadata set (Add. File 12). Empty values were filled as unknown.

### Metabolic versatility analysis

The metabolic versatility of all taxa was assessed by KEGG module completeness as determined by the MicrobeAnnotator (v2.0.5) pipeline (Ruiz-Perez et al. 2021) in light mode at default settings. The module completenesses were clustered using Ward's hierarchical clustering and plotted using in-house Python scripts. Optimal leaf ordering was enabled while plotting the clustermap.

### Pangenome construction and analysis

A pangenome was constructed for the full genome set using PPanGGOLiN (v2.0.5) (Gautreau et al. 2020) with gene defragmentation enabled. To account for the high diversity in this class-level genome set, clustering was carried out with a relaxed sequence identity threshold of 40% and an alignment coverage cutoff of 40%, while the pangenome was partitioned using a core genome threshold of 90% and an accessory genome threshold of 0.099% (or 1/1,010), as appeared most appropriate from our parameter sweep (Add. File 3). Presence/absence matrices for the different clostridial orders were extracted from the full class-level matrix by selecting for gene families present in at least one taxon from that order. The same pangenome partitioning thresholds were applied for consistency.

### Phylogeny construction

A phylogenetic tree for the full clostridial genome set was constructed with PhyloPhlAn (v3.0.67) (Asnicar et al. 2020) with a custom gene marker database derived from the representative protein sequences of the earlier identified core gene families. The tree was rooted using *B. subtilis* 168 as outgroup (RefSeq: GCF_000009045.1). The phylogeny was computed at medium diversity setting using DIAMOND (Buchfink et al. 2021) as the mapping algorithm, MAFFT (Katoh and Standley 2013) as the alignment algorithm, Trimal (Capella-Gutiérrez et al. 2009) as the alignment trimmer, and IQ-TREE2 (Minh et al. 2020) as the tree constructor. 10,000 ultrafast bootstraps (Minh et al. 2013) were requested by manually adding the “-bb 10000” flag to the IQ-TREE entry in the PhyloPhlAn configuration file. The outgroup was trimmed from the rooted phylogeny using the R package *ape*, after which the tree was visualized using iTol (v6.9) (Letunic and Bork 2024) and ggtree (v3.11.1) (Yu et al. 2017).

### Functional annotation of the pangenome

The protein family reference sequences of the pangenome were functionally annotated using eggNOG-mapper (v2.1.12) (Cantalapiedra et al. 2021). COG category counts and fractions were calculated using pandas (v2.2.1) and plotted using ggplot2 (v3.5.1).

### GD rate analysis

A GD rate analysis estimates the rate of GE within a clade and statistically compares these rates for significant differences. It requires the phylogenies of each examined clade to have an identical time scale. Therefore, a subtree was extracted for each order from the full phylogeny using *ape*. GE models were inferred for each order using *Panstripe* (Tonkin-Hill et al. 2023). All options were left at their defaults, except that Gaussian distributions were employed to fit the underlying GLMs. Cumulative GE plots, Uniform Manifold Approximation and Projection plots and statistical comparisons of the GE models were made using the appropriate *Panstripe* functions.

### Ancestral state reconstruction

To track the evolutionary origin of GEs, the ancestral states of all gene families were reconstructed from the full core genome phylogeny and the full pangenome presence/absence matrix. This was done according to Wagner parsimony with equal penalty scores for both gene gains and losses using Count (v10.4) (Csurös 2010). KOG annotations of the genes exchanged at the tree nodes of interest were retrieved from the earlier made eggNOG annotations and visualized using in-house R scripts.

### Mobilome analysis

Pseudogenes were detected using Pseudofinder (v1.1.0) (Syberg-Olsen et al. 2022) at default settings. The NCBI Protein database filtered for taxon ID 186801 (the *Clostridia* class) was used as reference database and indexed using DIAMOND (v2.0.14). Corrections for assembly fragmentation were not required, since pseudogene abundance showed only very weak correlation with both genome contiguity and N50 values (Add. File 2: Fig. S10). Insertion elements (ISEs) were inferred using ISEscan (v1.7.2.3) (Xie and Tang 2017) at default settings. BGCs were identified using antiSMASH (v8.0.0) (Blin et al. 2025) at default settings. Prophages were found by running PHASTEST (Wishart et al. 2023) in lite mode using the image from Docker Hub (digest 2477a2f3b6b8). Prophage host graphs were made using *supervenn* (v0.5.0). All MGE counts were normalized by dividing by the genome length. The normalized MGE abundances of each order were statistically compared using pairwise Brunner-Munzel tests with Bonferroni correction (*α* = 0.05) in scipy (v1.15.3). Significance annotations were added using *statannotations* (v0.6).

### BGC clustering analysis

All BGCs identified during the mobilome analysis were clustered using BiG-SCAPE (v1.1.8) (Navarro-Muñoz et al. 2020), adding MIBiG reference BGCs (flag “--mibig”), mixing all BGC classes (flag “--mix”), and including singletons (flag “--include_singletons”). A BiG-SCAPE distance cutoff value of 0.3 was applied as appeared most appropriate from a parameter sweep (Add. File 4) and the resulting similarity networks. To integrate phylogroup metadata in CytoScape (v3.10.2), the unidirectional network links in the output network files of BiG-SCAPE were annotated by order or MIBiG and converted into bidirectional links by adding the reverse link. BGC gene alignments were generated using clinker (v0.0.28) (Gilchrist and Chooi 2021).

## Supplementary Material

msag247_Supplementary_Data

## Data Availability

All genome assemblies analyzed in this study were retrieved from NCBI RefSeq. Accession codes and relevant metadata can be found in Add. File 1. All Supplementary material can also be found in the relevant folder at GitHub (https://github.com/LucoDevro/closHomer) and at Zenodo (https://doi.org/10.5281/zenodo.14719962).

## Code availability

The full analysis pipeline has been deposited in the relevant folder at GitHub (https://github.com/LucoDevro/closHomer) and at Zenodo (https://doi.org/10.5281/zenodo.14719962).
