# Supplementary material for: Comparative pangenomics unveils distinct host adaptation trends and conserved biosynthetic potential in microbiome Clostridia
Source: Mol Biol Evol. 2026 Oct 1;43(10):msag247. doi: 10.1093/molbev/msag247 (PMC13673991; doi:10.1093/molbev/msag247)
Supplement: Supplementary file 1 [file msag247_supplementary_data.zip › SupplementaryText1.pdf]

Supplementary Text 1: assessing the impact of taxonomic imbalance

In our study, we aimed to incorporate as much diversity as possible in our dataset. However, as the amount of available genomic data differs among clostridial orders, the resulting dataset is taxonomically imbalanced. Overrepresented lineages may therefore bias analysis outcomes and obscure diversity in other lineages.

This Supplementary Text assesses the robustness of our conclusions against different levels of imbalance. We applied different random subsampling regimes, ranging from the original dataset to strictly no taxonomic imbalance (i.e. no difference in sample size across the four orders). For each subsample, we retested for statistically significant differences in (1) core and tip genomic divergence rates, as well as (2) the three selected genomic hallmarks of progressive host adaptation (genome size, IS element counts, and pseudogene count). We added an effect size analysis as well to distinguish between genuine statistical signals lost due to excessive subsampling and spurious ones suppressed by removing imbalance bias.

Three random subsampling regimes with a step-wise increasing level of imbalance were tested (Table ***1***). In addition, we also tested a subsample with increased homogeneity in the *Lachnospirales* sample to eliminate the possibility of an artefact arising due to data heterogeneity in the most represented order. This subsample comprises its most coherent subgroup, as visible in the pangenome UMAP plot (**Figure 1**). For each random subsampling regime, five subsamples were taken. For each subsample, the pangenome construction, the comparative divergence rate analysis, and the statistical comparison of the genomic hallmarks abundance were re-executed. The effects in the effect size analysis were defined as an order's GD rate estimate or an MGE's normalised abundance. The effect size was calculated from the effects of the five random subsamples using approximate Hedges' *g* value as below, with M_i s_i^2, and n_i the average, the variance and the sample size of the divergence rate estimate of order *i*, respectively (Hedges, 1981).

$$g = \frac{M_1 - M_2}{\sqrt{\frac{s_1^2 + s_2^2}{2}}} \cdot \left(1 - \frac{3}{4(n_1 + n_2) - 9}\right)$$

Table 1. Order sample size for each subsampling regime. Different subsampling strengths have been tested to allow detecting statistical power loss due to excessive subsampling. A manual subsample with increased homogeneity in the *Lachnospirales* order was added to eliminate confounding heterogeneity effects.

Regime	*Clostridiales*	*Lachnospirales*	*Oscillospirales*	*Peptostreptococcales*
Original	209	349	164	94
Mild	209	209	164	94
Medium	164	164	164	94
Heavy	94	94	94	94
Homogenised	209	190	164	94

MGE abundance

Statistical significance remained stable across all examined subsamples, except for the ISE abundance (**Add. File 9**). In the heavy subsampling regime, with strict absence of taxonomic imbalance, the ISE abundance difference between *Lachnospirales* and *Oscillospirales* was not significantly different. However, the effect size analysis indicated that the original signal was likely genuine, as the effect size remained stable and Hedges' *g* value increased with milder subsampling, which is indicative of a stable signal and an improving signal-to-noise ratio, respectively. Significance persisted in the homogenised subsample as well, ruling out confounding heterogeneity effects (**Add. File 11**). Together with the other stably significant differences, we conclude from this that our findings regarding the host adaptation genomic hallmarks are robust for taxonomic imbalances.

GD rates

To challenge the divergence rate analyses, we focused on the strongest core and the strongest tip divergence rate effects: the core rate difference between *Lachnospirales* and *Oscillospirales*, and the tip rate difference between *Lachnospirales* and *Clostridiales*. In analogy with the ISE abundance, statistical significance tended to disappear in the heavier subsampling regimes with lower levels of sample imbalance (**Add. File 10**). Again, the effect size analysis indicated that the original signal was likely genuine, as the effect size remained stable across several random subsamples and the variance decreased when milder subsampling was applied, resulting in increasing Hedges' g values. In the homogenised subsample, significance persisted (**Add. File 11**). As a result, we conclude that, our original findings regarding the divergence rates hold as well.

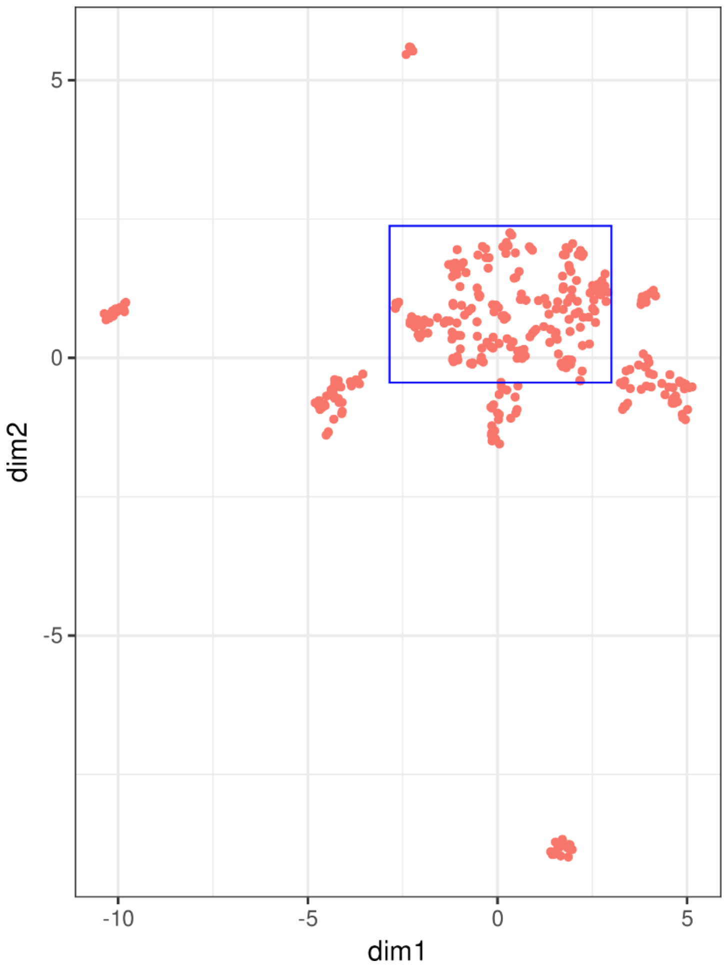

Figure 1. Pangenome UMAP plot of the *Lachnospirales* order. The blue rectangle indicates the subset selected as the homogenised *Lachnospirales* subsample.
